# 3-(3-Methoxy­benz­yl)-4-(2-methoxy­phen­yl)-1*H*-1,2,4-triazole-5(4*H*)-thione

**DOI:** 10.1107/S1600536808037215

**Published:** 2008-11-13

**Authors:** Muhammad Hanif, Ghulam Qadeer, Nasim Hasan Rama, Ales Ruzicka

**Affiliations:** a Department of Chemistry, Quaid-i-Azam University, Islamabad 45320, Pakistan; b Department of General and Inorganic Chemistry, Faculty of Chemical Technology, University of Pardubice, Nam. Cs. Legii’ 565, 53210 Pardubice, Czech Republic

## Abstract

In the title compound, C_17_H_17_N_3_O_2_S, the five-membered ring forms dihedral angles of 53.02 (3) and 78.57 (3)° with the 3-meth­oxy-substituted and 2-meth­oxy-substituted benzene rings, respectively. In the crystal structure, mol­ecules are linked into centrosymmetric dimers *via* inter­molecular N—H⋯S hydrogen bonds.

## Related literature

For background information on the biological activity of substituted triazole derivatives, see: Demirbas *et al.* (2002 ▶); Holla *et al.* (1998 ▶); Omar *et al.* (1986 ▶); Paulvannan *et al.* (2000 ▶); Turan-Zitouni *et al.* (1999 ▶); Kritsanida *et al.* (2002 ▶). For related structures, see: Öztürk *et al.* (2004*a* ▶,*b* ▶); Zhang *et al.* (2004 ▶). For bond-length data, see: Allen *et al.* (1987 ▶).
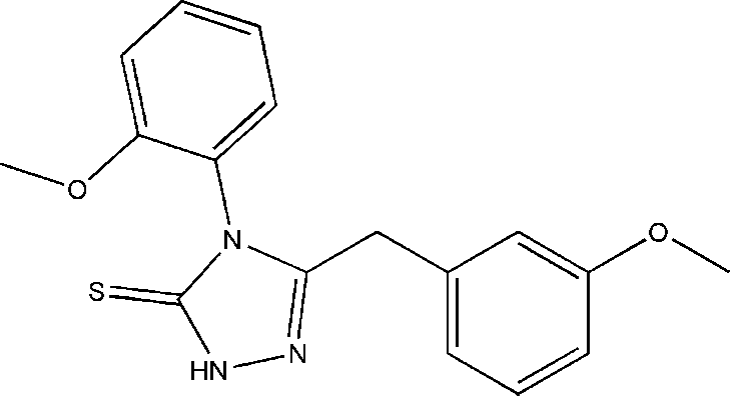


## Experimental

### 

#### Crystal data

C_17_H_17_N_3_O_2_S
*M* *_r_* = 327.40
Triclinic, 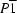

*a* = 7.3941 (3) Å
*b* = 10.6459 (5) Å
*c* = 12.1940 (8) Å
α = 68.841 (5)°
β = 74.317 (5)°
γ = 75.187 (5)°
*V* = 848.37 (8) Å^3^
*Z* = 2
Mo *K*α radiation
μ = 0.20 mm^−1^
*T* = 293 (2) K
0.40 × 0.24 × 0.15 mm

#### Data collection

Bruker–Nonius KappaCCD area-detector diffractometer
Absorption correction: integration (Gaussian; Coppens, 1970 ▶) *T*_min_ = 0.946, *T*_max_ = 0.983
10764 measured reflections
3708 independent reflections
2064 reflections with *I* > 2σ(*I*)
*R*_int_ = 0.079

#### Refinement

*R*[*F* ^2^ > 2σ(*F* ^2^)] = 0.073
*wR*(*F* ^2^) = 0.159
*S* = 1.10
3708 reflections
208 parameters
H-atom parameters constrained
Δρ_max_ = 0.32 e Å^−3^
Δρ_min_ = −0.24 e Å^−3^

### 

Data collection: *COLLECT* (Hooft, 1998 ▶) and *DENZO* (Otwin­owski & Minor, 1997 ▶); cell refinement: *DIRAX/LSQ* (Duisenberg, 1992 ▶); data reduction: *EVALCCD* (Duisenberg *et al.*, 2003 ▶); program(s) used to solve structure: *SIR92* (Altomare *et al.*, 1994 ▶); program(s) used to refine structure: *SHELXL97* (Sheldrick, 2008 ▶); molecular graphics: *PLATON* (Spek, 2003 ▶); software used to prepare material for publication: *SHELXL97*.

## Supplementary Material

Crystal structure: contains datablocks I, global. DOI: 10.1107/S1600536808037215/lh2729sup1.cif

Structure factors: contains datablocks I. DOI: 10.1107/S1600536808037215/lh2729Isup2.hkl

Additional supplementary materials:  crystallographic information; 3D view; checkCIF report

## Figures and Tables

**Table 1:** Hydrogen-bond geometry (Å, °)

*D*—H⋯*A*	*D*—H	H⋯*A*	*D*⋯*A*	*D*—H⋯*A*
N2—H2⋯S1^i^	0.86	2.42	3.277 (3)	172

Symmetry code: (i) 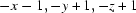.

**Fig. 1.**
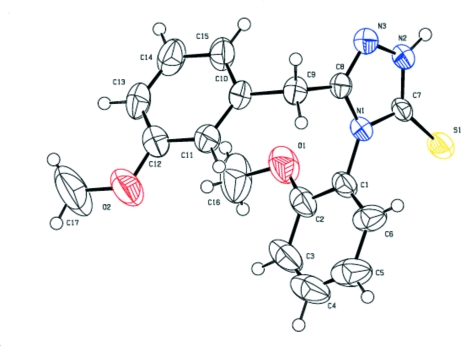
The molecular structure of (I) with 50% probability displacement ellipsoids (arbitrary spheres for H atoms).

**Fig. 2.**
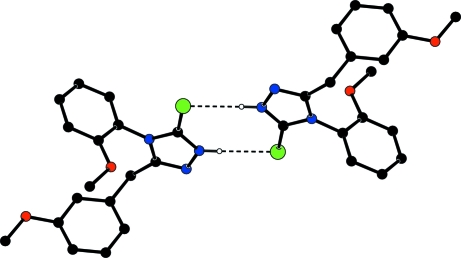
Part of the crystal structure of (I) showing hydrogen bonds as dashed lines.

**Fig. 3.**
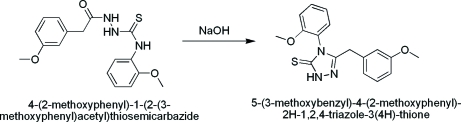
The reaction scheme.

## supplementary crystallographic information

### Comment

Substituted triazole derivatives display significant biological activity including antimicrobial (Holla *et al.*, 1998), analgesic (Turan-Zitouni *et al.*, 1999), antitumor (Demirbas *et al.*, 2002), antihypertensive (Paulvannan *et al.*, 2000) and antiviral activities (Kritsanida *et al.*, 2002). The biological activity is closely related to structure, possibly being due to the presence of the —N—C=S unit (Omar *et al.*, 1986). We are interested in the synthesis and biological activity of substituted triazole derivatives and report here the synthesis and crystal structure of the title compound, (I) (Fig. 1).

In the molecluar structure of (I), the bond lengths and angles are within normal ranges (Allen *et al.*, 1987) and comparable with those observed in related structures (Öztürk *et al.*, 2004*a*,b). The C7-S1 bond length [1.679 (3) A °] compares with 1.6773 (19) A ° in 4-(4-chlorophenyl)-3-(furan-2-yl)-1*H*-1,2,4-triazole-5(4*H*)-thione (Ozturk *et al.*, 2004*a*) and 1.668 (5) A ° in 4-amino-3-(1,2,3,4,5- pentahydroxypentyl)-1*H*-1,2,4-triazole-5(4*H*)-thione (Zhang *et al.*, 2004). In the triazole ring, the N3-C8 bond [1.294 (4)] bond shows the expected double bond character.

The rings A (N1—N3/C7/C8), B (C1—C6) and C (C10—C15) are eesentially planar and dihedral angles between them are A/B = 78.57 (3)°, A/C = 53.02 (3)° and B/C = 16.23 (3)°. In the crystal structure, molecules are linked into centrosymmetric dimers via intermolecular  N–H···S hydrogen bonds.

### Experimental

The synthesis of the title compound was carried out by refluxing a solution of 4-(2-methoxyphenyl)-1-(2-(3-methoxyphenyl)acetyl)thiosemicarbazide (3.45 g, 10 mmol) in 2 *M* NaOH for 5 h. Single crystals suitable for X-ray measurements were obtained by recrystallization from an aqeous ethanol solution at room temperature (yield: 75%; m.p. 469–470 K).

### Refinement

H atoms were placed in calculated positions with C-H = 0.93-0.97Å and N-H = 0.86Å and included in the refinement with U_iso_(H) = 1.2U_eq_(C,N). Although the atoms of substituted methoxy have larger than normal anisotropic displacement parameters, attempts to create disorder models did not improve the precision of the structure.

### Crystal data

**Table:**

C_17_H_17_N_3_O_2_S	*Z* = 2
*M**_r_* = 327.40	*F*(000) = 344
Triclinic, *P*1	*D*_x_ = 1.282 Mg m^−^^3^
Hall symbol: -P 1	Melting point: 469(1) K
*a* = 7.3941 (3) Å	Mo *K*α radiation, λ = 0.71073 Å
*b* = 10.6459 (5) Å	Cell parameters from 10808 reflections
*c* = 12.1940 (8) Å	θ = 1–27.5°
α = 68.841 (5)°	µ = 0.20 mm^−^^1^
β = 74.317 (5)°	*T* = 293 K
γ = 75.187 (5)°	Plate, colourless
*V* = 848.37 (8) Å^3^	0.40 × 0.24 × 0.15 mm

### Data collection

**Table:**

Bruker–Nonius KappaCCD area-detector diffractometer	3708 independent reflections
Radiation source: fine-focus sealed tube	2064 reflections with *I* > 2σ(*I*)
graphite	*R*_int_ = 0.079
Detector resolution: 9.091 pixels mm^-1^	θ_max_ = 27.5°, θ_min_ = 1.8°
φ and ω scans to fill the Ewald sphere	*h* = −9→9
Absorption correction: integration (Gaussian; Coppens, 1970)	*k* = −13→13
*T*_min_ = 0.946, *T*_max_ = 0.983	*l* = −15→15
10764 measured reflections	

### Refinement

**Table:**

Refinement on *F*^2^	Primary atom site location: structure-invariant direct methods
Least-squares matrix: full	Secondary atom site location: difference Fourier map
*R*[*F*^2^ > 2σ(*F*^2^)] = 0.073	Hydrogen site location: inferred from neighbouring sites
*wR*(*F*^2^) = 0.159	H-atom parameters constrained
*S* = 1.10	*w* = 1/[σ^2^(*F*_o_^2^) + (0.0364*P*)^2^ + 0.6393*P*] where *P* = (*F*_o_^2^ + 2*F*_c_^2^)/3
3708 reflections	(Δ/σ)_max_ < 0.001
208 parameters	Δρ_max_ = 0.32 e Å^−^^3^
0 restraints	Δρ_min_ = −0.24 e Å^−^^3^

### Special details

**Table:**

Geometry. All e.s.d.'s (except the e.s.d. in the dihedral angle between two l.s. planes) are estimated using the full covariance matrix. The cell e.s.d.'s are taken into account individually in the estimation of e.s.d.'s in distances, angles and torsion angles; correlations between e.s.d.'s in cell parameters are only used when they are defined by crystal symmetry. An approximate (isotropic) treatment of cell e.s.d.'s is used for estimating e.s.d.'s involving l.s. planes.
Refinement. Refinement of *F*^2^ against ALL reflections. The weighted *R*-factor *wR* and goodness of fit *S* are based on *F*^2^, conventional *R*-factors *R* are based on *F*, with *F* set to zero for negative *F*^2^. The threshold expression of *F*^2^ > σ(*F*^2^) is used only for calculating *R*-factors(gt) *etc*. and is not relevant to the choice of reflections for refinement. *R*-factors based on *F*^2^ are statistically about twice as large as those based on *F*, and *R*- factors based on ALL data will be even larger.

### Fractional atomic coordinates and isotropic or equivalent isotropic displacement parameters (Å^2^)

**Table:**

	*x*	*y*	*z*	*U*_iso_*/*U*_eq_	
S1	−0.37112 (12)	0.55979 (11)	0.30006 (8)	0.0515 (3)	
N2	−0.2330 (4)	0.4093 (3)	0.5026 (2)	0.0410 (7)	
H2	−0.3425	0.4150	0.5499	0.049*	
N1	−0.0166 (3)	0.4302 (3)	0.3462 (2)	0.0379 (6)	
N3	−0.0666 (4)	0.3402 (3)	0.5425 (2)	0.0444 (7)	
C7	−0.2087 (4)	0.4659 (3)	0.3847 (3)	0.0362 (7)	
C10	0.3280 (4)	0.1679 (3)	0.4006 (3)	0.0416 (8)	
C1	0.0831 (4)	0.4687 (4)	0.2239 (3)	0.0460 (9)	
C8	0.0625 (4)	0.3535 (3)	0.4452 (3)	0.0374 (7)	
C11	0.4677 (5)	0.1629 (4)	0.3017 (3)	0.0535 (9)	
H11	0.5259	0.2385	0.2566	0.064*	
O1	0.0235 (4)	0.2702 (3)	0.2152 (3)	0.0745 (8)	
C14	0.2995 (6)	−0.0616 (4)	0.4330 (4)	0.0700 (12)	
H14	0.2433	−0.1380	0.4789	0.084*	
C9	0.2686 (4)	0.2945 (3)	0.4391 (3)	0.0446 (8)	
H9A	0.2988	0.2723	0.5173	0.054*	
H9B	0.3424	0.3630	0.3829	0.054*	
C15	0.2434 (5)	0.0546 (4)	0.4662 (4)	0.0591 (11)	
H15	0.1477	0.0574	0.5333	0.071*	
C2	0.1052 (5)	0.3814 (5)	0.1581 (3)	0.0543 (10)	
C3	0.2031 (6)	0.4167 (6)	0.0396 (4)	0.0767 (15)	
H3	0.2209	0.3601	−0.0067	0.092*	
C13	0.4385 (6)	−0.0674 (4)	0.3336 (4)	0.0634 (11)	
H13	0.4743	−0.1460	0.3107	0.076*	
C6	0.1539 (5)	0.5894 (4)	0.1775 (3)	0.0605 (11)	
H6	0.1374	0.6459	0.2237	0.073*	
C12	0.5230 (6)	0.0434 (4)	0.2686 (4)	0.0639 (11)	
C5	0.2519 (6)	0.6227 (6)	0.0573 (4)	0.0808 (15)	
H5	0.3011	0.7034	0.0217	0.097*	
O2	0.6618 (5)	0.0512 (4)	0.1674 (3)	0.1103 (13)	
C16	0.0674 (8)	0.1673 (6)	0.1580 (5)	0.106 (2)	
H16A	0.0231	0.2046	0.0837	0.127*	
H16B	0.0055	0.0915	0.2094	0.127*	
H16C	0.2029	0.1366	0.1426	0.127*	
C4	0.2727 (6)	0.5350 (7)	−0.0064 (4)	0.0838 (17)	
H4	0.3387	0.5582	−0.0855	0.101*	
C17	0.7351 (12)	−0.0684 (7)	0.1341 (7)	0.181 (4)	
H17A	0.7803	−0.1401	0.2007	0.217*	
H17B	0.8390	−0.0520	0.0666	0.217*	
H17C	0.6375	−0.0952	0.1134	0.217*	

### Atomic displacement parameters (Å^2^)

**Table:**

	*U*^11^	*U*^22^	*U*^33^	*U*^12^	*U*^13^	*U*^23^
S1	0.0403 (5)	0.0619 (7)	0.0415 (5)	0.0027 (4)	−0.0018 (4)	−0.0162 (4)
N2	0.0386 (15)	0.0399 (17)	0.0390 (16)	−0.0030 (12)	0.0016 (12)	−0.0155 (13)
N1	0.0363 (14)	0.0392 (17)	0.0346 (15)	−0.0026 (12)	0.0005 (11)	−0.0154 (13)
N3	0.0486 (16)	0.0385 (17)	0.0425 (17)	−0.0052 (13)	−0.0046 (13)	−0.0135 (14)
C7	0.0415 (17)	0.0327 (19)	0.0339 (17)	−0.0070 (14)	0.0042 (13)	−0.0180 (15)
C10	0.0378 (17)	0.035 (2)	0.052 (2)	0.0009 (14)	−0.0134 (15)	−0.0141 (16)
C1	0.0340 (17)	0.059 (2)	0.0374 (18)	0.0044 (16)	−0.0034 (14)	−0.0182 (18)
C8	0.0442 (18)	0.0298 (18)	0.0399 (19)	−0.0055 (14)	−0.0060 (15)	−0.0152 (15)
C11	0.063 (2)	0.039 (2)	0.054 (2)	−0.0098 (17)	−0.0022 (18)	−0.0157 (18)
O1	0.086 (2)	0.076 (2)	0.076 (2)	−0.0040 (17)	−0.0174 (16)	−0.0474 (18)
C14	0.062 (2)	0.041 (2)	0.099 (3)	−0.0095 (19)	−0.010 (2)	−0.017 (2)
C9	0.0454 (19)	0.039 (2)	0.050 (2)	−0.0018 (15)	−0.0128 (16)	−0.0164 (17)
C15	0.046 (2)	0.044 (2)	0.077 (3)	−0.0036 (18)	0.0007 (19)	−0.020 (2)
C2	0.040 (2)	0.075 (3)	0.047 (2)	0.0079 (19)	−0.0101 (16)	−0.030 (2)
C3	0.049 (2)	0.124 (5)	0.050 (3)	0.015 (3)	−0.007 (2)	−0.041 (3)
C13	0.072 (3)	0.040 (2)	0.082 (3)	0.003 (2)	−0.022 (2)	−0.029 (2)
C6	0.041 (2)	0.068 (3)	0.053 (2)	−0.0121 (19)	−0.0062 (17)	0.003 (2)
C12	0.066 (3)	0.057 (3)	0.062 (3)	−0.002 (2)	0.000 (2)	−0.027 (2)
C5	0.052 (2)	0.102 (4)	0.061 (3)	−0.021 (2)	−0.005 (2)	0.007 (3)
O2	0.142 (3)	0.076 (2)	0.094 (3)	−0.024 (2)	0.042 (2)	−0.049 (2)
C16	0.129 (5)	0.094 (4)	0.130 (5)	0.021 (3)	−0.062 (4)	−0.077 (4)
C4	0.047 (2)	0.137 (5)	0.047 (3)	−0.002 (3)	0.000 (2)	−0.020 (3)
C17	0.222 (8)	0.116 (6)	0.175 (7)	−0.045 (5)	0.100 (6)	−0.102 (6)

### Geometric parameters (Å, °)

**Table:**

S1—C7	1.679 (3)	C9—H9A	0.9700
N2—C7	1.324 (4)	C9—H9B	0.9700
N2—N3	1.377 (4)	C15—H15	0.9300
N2—H2	0.8600	C2—C3	1.390 (6)
N1—C7	1.369 (4)	C3—C4	1.353 (7)
N1—C8	1.375 (4)	C3—H3	0.9300
N1—C1	1.434 (4)	C13—C12	1.358 (6)
N3—C8	1.294 (4)	C13—H13	0.9300
C10—C11	1.367 (5)	C6—C5	1.407 (6)
C10—C15	1.380 (5)	C6—H6	0.9300
C10—C9	1.507 (5)	C12—O2	1.366 (5)
C1—C6	1.380 (5)	C5—C4	1.372 (7)
C1—C2	1.387 (5)	C5—H5	0.9300
C8—C9	1.485 (4)	O2—C17	1.407 (6)
C11—C12	1.402 (5)	C16—H16A	0.9599
C11—H11	0.9300	C16—H16B	0.9601
O1—C2	1.340 (5)	C16—H16C	0.9600
O1—C16	1.428 (5)	C4—H4	0.9300
C14—C15	1.370 (5)	C17—H17A	0.9600
C14—C13	1.370 (6)	C17—H17B	0.9600
C14—H14	0.9299	C17—H17C	0.9599
			
C7—N2—N3	113.7 (3)	O1—C2—C1	115.7 (3)
C7—N2—H2	123.2	O1—C2—C3	125.5 (4)
N3—N2—H2	123.1	C1—C2—C3	118.7 (5)
C7—N1—C8	108.1 (2)	C4—C3—C2	118.4 (5)
C7—N1—C1	125.4 (3)	C4—C3—H3	120.8
C8—N1—C1	126.5 (2)	C2—C3—H3	120.8
C8—N3—N2	103.9 (3)	C12—C13—C14	119.2 (4)
N2—C7—N1	103.5 (3)	C12—C13—H13	120.3
N2—C7—S1	129.2 (2)	C14—C13—H13	120.5
N1—C7—S1	127.3 (2)	C1—C6—C5	116.9 (5)
C11—C10—C15	119.2 (3)	C1—C6—H6	121.3
C11—C10—C9	120.7 (3)	C5—C6—H6	121.7
C15—C10—C9	120.1 (3)	C13—C12—O2	124.9 (4)
C6—C1—C2	123.1 (4)	C13—C12—C11	120.6 (4)
C6—C1—N1	118.8 (3)	O2—C12—C11	114.5 (4)
C2—C1—N1	118.1 (4)	C4—C5—C6	119.1 (5)
N3—C8—N1	110.8 (3)	C4—C5—H5	120.7
N3—C8—C9	125.4 (3)	C6—C5—H5	120.1
N1—C8—C9	123.8 (3)	C12—O2—C17	117.6 (4)
C10—C11—C12	119.8 (4)	O1—C16—H16A	109.5
C10—C11—H11	120.1	O1—C16—H16B	109.4
C12—C11—H11	120.1	H16A—C16—H16B	109.5
C2—O1—C16	117.5 (4)	O1—C16—H16C	109.5
C15—C14—C13	120.9 (4)	H16A—C16—H16C	109.5
C15—C14—H14	119.6	H16B—C16—H16C	109.5
C13—C14—H14	119.5	C3—C4—C5	123.7 (5)
C8—C9—C10	113.6 (3)	C3—C4—H4	118.3
C8—C9—H9A	108.9	C5—C4—H4	118.1
C10—C9—H9A	108.9	O2—C17—H17A	108.6
C8—C9—H9B	108.8	O2—C17—H17B	109.5
C10—C9—H9B	108.7	H17A—C17—H17B	109.5
H9A—C9—H9B	107.7	O2—C17—H17C	110.4
C14—C15—C10	120.4 (4)	H17A—C17—H17C	109.5
C14—C15—H15	119.9	H17B—C17—H17C	109.5
C10—C15—H15	119.7		

### Hydrogen-bond geometry (Å, °)

**Table:**

*D*—H···*A*	*D*—H	H···*A*	*D*···*A*	*D*—H···*A*
N2—H2···S1^i^	0.86	2.42	3.277 (3)	172.

Symmetry codes: (i) −*x*−1, −*y*+1, −*z*+1.
